# The medicalization of disease as a factor of abandonment and dissatisfaction with HIV treatment services

**DOI:** 10.1186/1742-4690-9-S1-P91

**Published:** 2012-05-25

**Authors:** Carmen Rodríguez Reinado, Teresa Blasco, Hernández Jesus, Nzang Esono

**Affiliations:** 1 University of Huelva, Huelva, Spain

## Introduction

Medicalization of the disease is a social phenomenon not exclusive of industrialized societies. In countries with medium and low human development indexes and developing economies, this process is prevailing due to the expansion of the biomedical model. This is the case of many African countries and more concretely in Equatorial Guinea.

## Methodology

Qualitative research, based on the application of semi-structured interview (30) as the technique for gathering information. Ambit: Bata, Equatorial Guinea. Purposeful sampling: homogeneous type by subgroup; 1. HIV-positive people who abandoned diagnostic and treatment services. 2. HIV-positive people who are utilising HIV treatment services. Location: General Hospital and Outpatient Treatment Centers. Unit of analysis: utilization of diagnostic and treatment services.

## Method of analysis

Grounded Theory Method. Data triangulation: internal by two observers and theoretical.

## Results

- Drugs are a central topic on the discourse about the disease.

- Regardless of the population profile, all the informants share a medicalized view of HIV and its treatment

- In the asymptomatic phase of HIV, avoiding to prescribe drugs reinforces the process of non-acceptance of the disease leading the patient to question his positive diagnosis.

- Within the profile of HIV-positive respondents still in treatment, medication is the element of the care process that takes on more importance. Avoiding to prescribe drugs is an element of dissatisfaction with health services.

- In the profile of HIV-positive respondents who abandoned HIV diagnostic and treatment services, no prescription drugs was one of the reasons for abandonment.

## Conclusions

The medicalization of the disease has affected the social construction of HIV as a disease and is one of the reasons for abandonment and dissatisfaction with the care received in diagnostic and treatment centers. It therefore represents a factor for intervention and to modify in order to reduce the rates of abandonment for such services.

